# Endoscopic lateral transorbital approach for resection of a middle fossa meningioma

**DOI:** 10.3171/2025.1.FOCVID24176

**Published:** 2025-04-01

**Authors:** Juan L. Gómez-Amador, Rodolfo Villalobos-Díaz, Michel G. Mondragón-Soto, Ricardo Marian-Magaña, Jorge F. Aragón-Arreola, Luis A. Rodriguez-Hernandez

**Affiliations:** Department of Neurosurgery, National Institute of Neurology and Neurosurgery "Manuel Velasco Suárez," Mexico City, Mexico

**Keywords:** endoscopic transorbital, skull base surgery, endoscopic surgery, meningioma

## Abstract

The lateral transorbital approach (LTOA) is useful for lesions situated in the middle fossa, adjacent to the lateral wall of the cavernous sinus, as it minimizes brain retraction and provides an extradural corridor. In this video, the authors present the case of a 35-year-old male, complaining of headache and diplopia. MRI revealed a dural-based middle fossa lesion adjacent to the lateral wall of the cavernous sinus. An endoscopic LTOA was performed through a superior eyelid incision. Postoperative MRI showed minimal residual enhancement, and histopathological analysis reported a grade III anaplastic meningioma. The patient was discharged after 48 hours without a new neurological deficit.

The video can be found here: https://stream.cadmore.media/r10.3171/2025.1.FOCVID24176

## Transcript

In this video, we discuss the technical nuances of an endoscopic lateral transorbital approach for the resection of a middle fossa meningioma.

This is the case of a 35-year-old male with no relevant past medical history who presented with headache and diplopia of the right eye. Physical examination was only relevant for right eye exophthalmos. The contrast MRI revealed a middle fossa mass located medial to the temporal lobe and adjacent to the lateral wall of the right cavernous sinus.

### 0:54 Clinical Setup.

After discussing the management options with the patient, the team decided to perform a surgical resection through a lateral transorbital approach. The patient was positioned supine, and a three-pin head fixation device was applied. Cranial navigation was utilized to assist with the localization and orientation of the surgical approach. A 4-mm, 0° rigid endoscope, was utilized for visualization throughout the procedure, held by the assistant.

### 1:23 Surgical Procedure.

First, a superior eyelid crease incision was made, and the periorbital tissue was dissected. The periorbital tissue was gently retracted medially to expose the greater sphenoid wing, which constitutes the lateral wall of the orbit.^1,2^ Then the bone was drilled with a 3-mm diamond burr, first inferiorly along the greater sphenoid wing and then superiorly at the frontal roof until reaching the lesser sphenoid wing. This lesser sphenoid wing was then removed using Kerrison rongeurs. Finally, the sagittal crest at the most medial aspect of the approach was resected to establish communication between the surgical corridor and the superior orbital fissure.^3–5^ The meningo-orbital band was identified, coagulated, and divided. Then the intradural dissection was initiated at this point and the external dural layer was peeled away from the lateral wall of the cavernous sinus.^6^ Medial disconnection of the tumor was achieved with this maneuver.

### 2:33 Removal of Tumor.

The dura was opened in a linear fashion, and immediately, the tumor was identified. The resection was performed using a bimanual technique, carefully dissecting the tumor from the surrounding structures. Given the consistency of the tumor, an initial central debulking was performed, allowing it to be extracted in a piecemeal fashion. Great care was taken to avoid excessive traction on the surrounding tissues during the resection. Following initial central debulking, the tumor capsule was carefully mobilized through blunt dissection and resected when feasible. The pituitary curettes were used to carefully peel away any remnants attached to the dura. The surgical cavity was thoroughly irrigated, and hemostasis was achieved with bipolar cautery and hemostatic agents.

### 3:26 Reconstruction.

Reconstruction was performed in a multilayered fashion, using artificial dural graft, which was placed both in an inlay and an onlay fashion. No fat graft was placed in the surgical cavity, as the support provided by the orbital structures was believed to be sufficient to prevent a cerebrospinal fluid leak.^4^ The retractors were removed, and the eyelid was closed with a subdermal running suture.

### 3:50 Postoperative Course.

Postoperative MRI revealed minimal residual enhancement, and histopathological analysis reported a grade III meningioma, prompting referral of the patient to radiosurgery for adjuvant treatment. The patient expressed satisfaction with both the surgical and cosmetic outcomes of the operation.

## Disclosures

The authors report no conflict of interest concerning the materials or methods used in this study or the findings specified in this publication.

## Author Contributions

Primary surgeon: Gómez-Amador. Assistant surgeon: Villalobos-Díaz, Marian-Magaña, Aragón-Arreola, Rodriguez-Hernandez. Editing and drafting the video and abstract: Villalobos-Díaz, Mondragón-Soto, Marian-Magaña, Aragón-Arreola. Critically revising the work: Villalobos-Díaz, Gómez-Amador, Mondragón-Soto, Aragón-Arreola, Rodriguez-Hernandez. Reviewed submitted version of the work: Villalobos-Díaz, Gómez-Amador, Mondragón-Soto, Marian-Magaña, Rodriguez-Hernandez. Approved the final version of the work on behalf of all authors: Villalobos-Díaz, Supervision: Gómez-Amador, Mondragón-Soto, Marian-Magaña, Aragón-Arreola.

## Supplemental Information

### Patient Informed Consent

The necessary patient informed consent was obtained in this study.
